# Discovery of Novel Angiotensin-Converting Enzyme Inhibitory Peptides from *Todarodes pacificus* and Their Inhibitory Mechanism: In Silico and In Vitro Studies

**DOI:** 10.3390/ijms20174159

**Published:** 2019-08-26

**Authors:** Dingyi Yu, Cong Wang, Yufeng Song, Junxiang Zhu, Xiaojun Zhang

**Affiliations:** 1 Laboratory of Aquatic Product Processing and Quality Safety, Marine Fisheries Research Institute of Zhejiang, Zhoushan 316021, China; 2 Guangxi Key Laboratory of Chemistry and Engineering of Forest Products, Key Laboratory of Guangxi Colleges and Universities for Food Safety and Pharmaceutical Analytical Chemistry, School of Chemistry and Chemical Engineering, Guangxi University for Nationalities, Nanning 530006, China

**Keywords:** ACE inhibitory peptides, *Todarodes pacificus*, molecular docking, molecular dynamics

## Abstract

In order to rapidly and efficiently excavate antihypertensive ingredients in *Todarodes* *pacificus*, its myosin heavy chain was hydrolyzed in silico and the angiotensin-converting enzyme (ACE) inhibitory peptides were predicted using integrated bioinformatics tools. The results showed the degree of hydrolysis (*DH*) theoretically achieved 56.8% when digested with papain, ficin, and prolyl endopeptidase (PREP), producing 126 ACE inhibitory peptides. By predicting the toxicity, allergenicity, gastrointestinal stability, and intestinal epithelial permeability, 30 peptides were finally screened, of which 21 had been reported and 9 were new. Moreover, the newly discovered peptides were synthesized to evaluate their in vitro ACE inhibition, showing Ile-Ile-Tyr and Asn-Pro-Pro-Lys had strong effects with a pIC_50_ of 4.58 and 4.41, respectively. Further, their interaction mechanisms and bonding configurations with ACE were explored by molecular simulation. The preferred conformation of Ile-Ile-Tyr and Asn-Pro-Pro-Lys located in ACE were successfully predicted using the appropriate docking parameters. The molecular dynamics (MD) result indicated that they bound tightly to the active site of ACE by means of coordination with Zn(II) and hydrogen bonding and hydrophobic interaction with the residues in the pockets of S_1_ and S_2_, resulting in stable complexes. In summary, this work proposed a strategy for screening and identifying antihypertensive peptides from *Todarodes* *pacificus*.

## 1. Introduction

The angiotensin-converting enzyme (ACE) is a metal carboxypeptidase involved in the renin-angiotensin system, which not only controls blood pressure and electrolyte homeostasis, but also renal function and myocardial remodeling [1]. It can convert the hormone, angiotensin I, to angiotensin II, which is a potent vasoconstrictive peptide that causes blood vessels to narrow, resulting in an elevated blood pressure [2]. Therefore, ACE is a major target for lowering blood pressure and cardiovascular therapies, and its inhibition mainly leads to an overall antihypertensive effect. 

ACE inhibitors, such as captopril, lisinopril, enalapril, and ramipril, have been on the market for more than three decades [1]. Although they have a tight binding pattern with the generic ACE active sites (Zn^2+^, S_1_, S_1_’, and S_2_’ pockets) and exhibit excellent cardiovascular therapeutic capabilities, ACE inhibitors have numerous side effects that can impair renal function, cause hyperkalemia [3], and develop angioedema [4]. Recently, a population-based cohort study has revealed a relationship between the long-term use of ACE inhibitors and an increased risk of lung cancer [5]. Their side effects have focused researchers’ interest on the search for alternative non-toxic and naturally generated peptides for controlling blood pressure. 

Almost accompanied by the development of antihypertensive drugs, food-derived peptides with antihypertensive properties have received great attention over the past 30 years [6]. The antihypertensive effects of some well-known food-derived peptides are mediated by the inhibition of ACE. Compared with synthetic drugs, these natural ACE inhibitors are considered milder and safer. Many studies have focused on identifying new antihypertensive sequences from different food sources including milk, cheese, meat, various plants, and algae [6]. The tripeptide Ile-Pro-Pro and Val-Pro-Pro are released from *β*- and *κ*-casein produced by the fermentation of *Lactobacillus helveticus* and *Sacharomyces cerevisiae* on milk [7]. In the follow-up research progress, it was also confirmed that Ile-Pro-Pro and Val-Pro-Pro attenuate the development of hypertension in spontaneously hypertensive rats and has hypotensive properties in hypertensive subjects [8,9]. Other effective dipeptides or tripeptides, which were originally discovered from different edible sources, such as pork, chicken, and eggs, namely Ile-Trp, Leu-Trp, Phe-Pro, Phe-Tyr, Ile-Pro-Ala, Gly-Lys-Pro, Val-Tyr-Pro, Tyr-Pro, and Val-Tyr, also have the similar ACE inhibitory activity. In addition, the current studies have found ACE inhibitory peptides in a variety of marine protein resources, including fish, cephalopods, bivalves, algae, and some processing by-products [10,11,12]. The types and quantities of these proteins are much larger than terrestrial resources and have not been well developed. Furthermore, due to the specificity of the marine environment, their protein composition is different from terrestrial biological proteins. Therefore, proteins from the ocean can serve as a potential resource for excavating ACE inhibitory peptides.

The entire process of preparing bioactive peptides from natural proteins by conventional methods is cumbersome and time consuming, thus limiting and delaying their use in food and pharmaceutical applications [13]. Alternatively, with the development of computer technology and the popularity of various online databases, the prediction and identification of food protein-derived bioactive peptides has become a feasible method [14]. Through this approach in silico, researchers can release potential bioactive peptides from native protein sequences using one or several proteases of choice. Nowadays, some ACE inhibitory peptides have been identified from many plants and animals in silico, such as wheat [15], scallop [16], meat proteins [17], and common oat [18]. Although the screening process for these jobs varies from one standard to another, they all showed fast and efficient features. 

In some Asian countries, *Todarodes pacificus* is a popular seafood because of its unique flavor and taste as well as its rich quality protein [19]. It is also one of the most important commercial cephalopods in China. A previous study demonstrated that the enzymatic hydrolysates of *Todarodes pacificus* exhibited higher antioxidant, tyrosinase inhibitory, and antielastase activities [20]. However, reports on the development of ACE inhibitory peptides in it are rare. So far, some literatures have only reported the extraction of ACE inhibitory peptides from the other species of squids. For instance, Lin et al. prepared the pepsin hydrolysate (<2 kDa) of *Dosidicus eschrichitii* with a great ACE inhibitory activity in vitro (IC_50_ = 0.33 mg/mL) [21]. Similarly, Alemán et al. reported the Alcalase hydrolysate from the inner and outer tunics of *Dosidicus gigas*, which was a potent ACE inhibitor (IC_50_ = 0.34 mg/mL) [22]. They also identified a new ACE inhibitory decapeptide (Gly-Arg-Gly-Ser-Val-Pro-Ala-Hyp-Gly-Pro) from the skin of *Dosidicus gigas* using pepsin and pancreatin digestion [23].

Therefore, the aim of this study was to explore the ACE inhibitory peptide from *Todarodes pacificus* based on a computer-assisted method. Plant proteases and prolyl endopeptidase (PREP) were selected to hydrolyze the myosin heavy chain of *Todarodes pacificus* in silico. Subsequently, the ACE inhibitory peptides therein were predicted and screened according to toxicity, allergenicity, gastrointestinal stability, and intestinal epithelial permeability. Among the peptides finally obtained, the ACE inhibitory activity of those newly discovered was evaluated by an in vitro method. Finally, the interactions mechanism between ACE and some new peptides were studied via molecular docking and molecular dynamic (MD) simulation. This study is also an attempt to provide a protocol for the screening of ACE inhibitory peptides from food source proteins.

## 2. Results and Discussion

### 2.1. In Silico Hydrolysis of the Myosin Heavy Chain of Todarodes Pacificus

In the present study, two proteases derived from plants were used to hydrolyze the myosin heavy chain of *Todarodes pacificus* to produce ACE inhibitory peptides. They showed a wider specificity in comparison with other enzymes such as trypsin or pepsin, cleaving peptide bonds from multiple regions and frequently acting as both exo- and endopeptidase [24]. Besides, another enzyme PREP was selected for a supplementary hydrolysis after the action of plant proteases, which cleaved peptide bonds at the C-terminal side of Pro residues [25]. This hydrolysis specificity played a key role in the release of potent ACE inhibitory peptides, because a common feature of many potent ACE inhibitory sequences was the presence of Pro residues at one or more positions of the C-terminal region [26].

**Figure 1:**
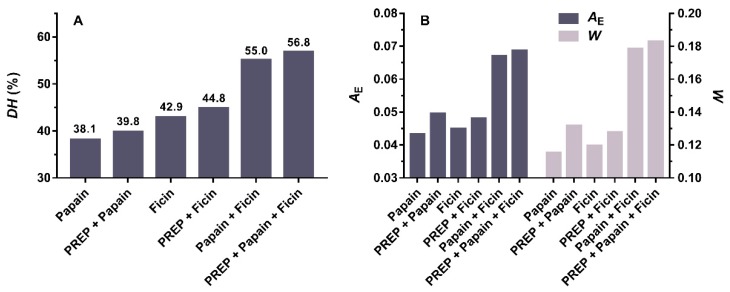
Degrees of hydrolysis (*DH*), release frequency (*A*_E_), and relative frequency (*W*) of the myosin heavy chain of *Todarodes pacificus* digested by different enzymes. PREP represented prolyl endopeptidase.

After digestion using the BIOPEP-UWM online [27], the simulated degree of hydrolysis (*DH)* of all enzymatic treatments is shown in Figure 1A. The result showed the papain, ficin, and their combination for the myosin heavy chain of *Todarodes pacificus* had the *DH* value of 38.1%, 42.9%, and 55.0%, respectively. After addition of PREP, the *DH* of each group increased, from 38.1% to 39.8% for papain, and from 42.9% to 47.9% for ficin. The *DH* of the complex enzymes consisting of papain, ficin and PREP was the highest in all enzyme treatments, reaching 56.8%. The above results indicated that the introduction of PREP was advantageous for the hydrolysis of the myosin heavy chain from *Todarodes pacificus*.

Figure 1B showed the changes in frequency (*A*_E_) and relative frequency (*W*) of the myosin heavy chain of *Todarodes pacificus* digested by different enzymes. *A*_E_ was the ratio of the number of ACE inhibitory peptides released by a given enzyme to the number of amino acid residues of the myosin [27]. *W* was the ratio of the number of ACE inhibitory peptides released by a given enzyme to the number of ACE inhibitory peptides in the myosin sequence [27]. They were all the quantitative parameters describing the predicted efficiency of proteolysis. The data in Figure 1B showed that the combination of papain and ficin significantly increased the *A*_E_ and *W* compared to hydrolysis using a single enzyme. Some previous studies indicated that papain and ficin were commonly used to produce antihypertensive peptides from food materials, such as oat [18], crude barley [28], and bovine fibrinogen [29]. In this study, papain and ficin were combined for enzymatic hydrolysis, indicating that it has a greater potential to release ACE inhibitory peptide than when they were used alone. After myosin underwent supplemental hydrolysis by PREP, the *A*_E_ and *W* further increased for all enzymatic treatments. For the case of single digestion by papain, supplementation with PREP increased *A*_E_ and *W* by 14.3% and 14.1%, respectively, which was the largest increase in all hydrolysis groups. For the complex group of papain and ficin, the addition of PREP slightly enhanced the acquired frequency of ACE inhibitory peptides. The above results illustrated that the hydrolysis of squid myosin by papain and ficin together could enhance the effect of hydrolysis and improve the efficiency of release of potential antihypertensive peptides in the myosin heavy chain of *Todarodes pacificus*.

### 2.2. In Silico Prediction of ACE Inhibitory Peptides from Myosin Heavy Chain of Todarodes Pacificus

**Figure 2:**
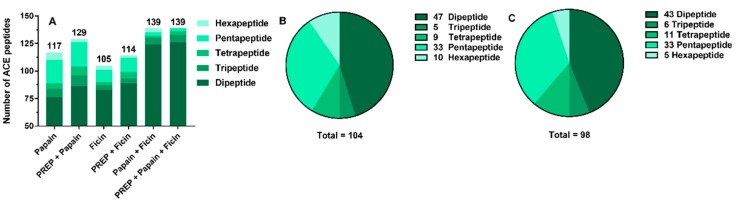
Number of ACE inhibitory peptides produced by different proteases in silico (**A**). Species of ACE inhibitory peptides released by hydrolysis using plant proteases without PREP (**B**) and with PREP (**C**) in silico. PREP was prolyl endopeptidase.

As discussed above, peptides with ACE inhibitory activity could be produced from the myosin heavy chain of *Todarodes pacificus* by two plant proteases and the supplemented PREP. However, since the ACE inhibitory peptides identified by the BIOPEP-UWM database were both dip- and tripeptides in this study, the ACE inhibitory tetra-, penta-, and hexapeptides obtained by digestion with papain, ficin, and PREP were further predicted using the AHTpin database [30]. Then, the number and type of ACE inhibiting peptides were statistically analyzed. As shown in Figure 2A, 117 and 105 ACE inhibitory peptides were produced by papain and ficin hydrolysis, respectively. Compared to the single enzyme treatment, the combination of papain and ficin yielded more ACE inhibitory peptides. When PREP was introduced into a single plant protease, the total number of ACE inhibitory peptides also increased, for papain from 117 to 129 and for ficin from 105 to 114. Additionally, comparing the hydrolysis of papain–ficin and papain–ficin–PREP, the number of ACE inhibitory peptides remained unchanged, both of which were 139 peptides. It was worth noting that after the myosin heavy chain of *Todarodes pacificus* was digested with papain and PREP, the number of di- and tripeptides increased by 10.2%, which was significantly higher than that of ficin and papain–ficin after adding PREP, well agreeing with the data of *A*_E_ and *W* (Figure 1B). 

After merging the peptides with repeat sequences, the kinds of ACE inhibitory peptides obtained by the hydrolysis of plant proteases and plant proteases followed by PREP were analyzed and plotted in Figure 2B,C. The results showed that the addition of PREP to myosin digestion reduced the types of ACE inhibitory peptides, from 104 to 98. In detail, the di- and hexapeptides were reduced, and the tri- and tetrapeptides were increased. 

The di- and tripeptides with ACE inhibitory activity generated by plant proteases without PREP are summarized in Table S1. They were all known peptides reported in some previous studies. Their pIC_50_ values were provided by the databases of AHTpin and BIOPEP-UWM, and their sources could be found in the databases of BIOPEP-UWM [27] and AHTPDB [31]. Among them, Met-Phe, Ile-Tyr, Asp-Gly, Glu-Tyr, Ser-Thr, and Val-Phe showed higher pIC_50_ values of 6.04, 5.68, 5.67, 5.57, 5.39, and 5.04, respectively. After the addition of PREP, as summarized in Table S2, the three groups of proteases produced 49 di- and tripeptides, of which the disappeared peptides were Ala-Phe, Glu-Trp, Glu-Tyr, Ile-Tyr, Met-Gly, Met-Tyr, Asn-Gly, Asn-Tyr, Pro-Gly, Pro-His, Pro-Leu, Pro-Pro-Lys, Val-Ala-Phe, Val-Glu, and Trp-Leu. The newly formed peptides were Ala-Ile-Pro, Ala-Pro, Ala-Val-Pro, Glu-Gly, Ile-Tyr, Lys-Pro, Met-Gly, Met-Tyr, Thr-Pro, and Val-Pro. Subsequently, the 52 tetra-, penta-, and hexapeptides with ACE inhibitory activity in Table S3 were obtained by plant proteases without PREP and predicted by the AHTpin webserver [30]. Among them, Pro-Ile-Tyr-Thr, Glu-Lys-Ser-Arg, Ala-Ile-Asn-Pro-Tyr-Arg, Val-Ile-Gln-Tyr, Glu-Pro-Ile-Val-Lys, and Gln-Glu-Gln-Asp-His received higher scores of 1.06, 1.00, 0.99, 0.91, 0.87, and 0.86, respectively. After adding PREP, the proteases produced a total of 49 ACE inhibitory tetra-, penta-, and hexapeptides (Table S4). Among them, 19 peptides disappeared, and 16 peptides were newly produced, some of which had a C-terminal Pro residue, including Ala-Glu-Met-Pro-Pro, Ala-Ile-Asn-Pro, Cys-Ile-Ala-Ile-Asn-Pro, Cys-Ile-Ile-Pro, Ile-Glu-Lys-Pro, Asn-Ala-Ile-Pro, Asn-Cys-Trp-Val-Pro, Asn-Lys-Val-Lys-Pro, Gln-Cys-Asn-Pro, Gln-Met-Asn-Pro-Pro, and Ser-Lys-Glu-Pro. Moreover, Ile-Glu-Lys-Pro was the known ACE inhibitory peptide derived from catfish myofibrillar protein with an IC_50_ value of 2.1 μg/mL [32].

### 2.3. In Silico Screening of ACE Inhibitory Peptides from Myosin Heavy Chain of Todarodes pacificus

As described above, 104 and 98 ACE inhibitory peptides derived from *Todarodes pacificus* were obtained by plant proteases with and without PREP, respectively. Then, the peptides with the same sequence in the above two groups were merged, producing a total of 126 peptides for further screening based on the prediction of toxicity, allergenicity, gastrointestinal stability, and intestinal epithelial permeability.

**Figure 3:**
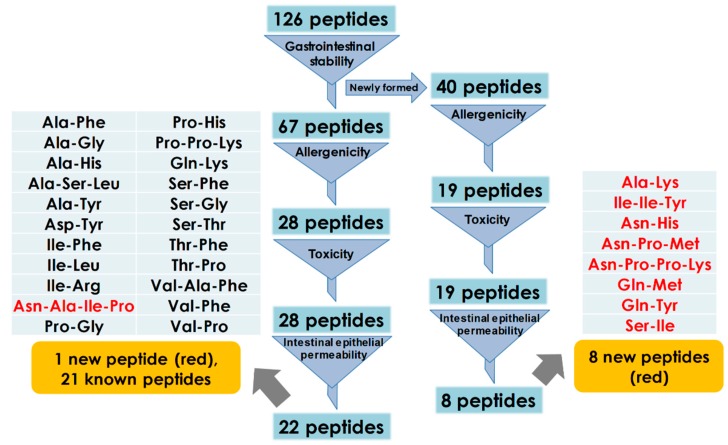
Screening process for ACE inhibitory peptides based on toxicity, allergenicity, gastrointestinal stability, and intestinal epithelial permeability.

As shown in Figure 3, the ACE inhibitory peptides that were resistant to the gastrointestinal environment were screened first. This was important because the active potential of peptides depended on their ability to access their target sites in an active form after passage through the gastrointestinal tract [6]. For example, Lys-Val-Leu-Pro-Val-Pro-Gln from the β-casein fragment showed a relatively weak ACE inhibitory activity in vitro while its antihypertensive effect in spontaneously hypertensive rats was effective. This was due to the conversion of Lys-Val-Leu-Pro-Val-Pro-Gln to Lys-Val-Leu-Pro-Val-Pro by pancreatin in the gastrointestinal tract, which was an active form in vivo [33]. The results in Figure 3 showed that 67 of the ACE inhibiting peptides were resistant to pepsin, trypsin, and chymotrypsin. The remaining 59 peptides were digested by the gastrointestinal proteases to form 40 new peptides. Together with the above-described 67 peptides resistant to gastrointestinal digestion, they were subjected to subsequent allergic prediction.

Immunogenicity and toxicity remain the thorny issues in the development of peptide-based drugs. So, the potential allergenicity of the above gastrointestinally stable peptides was assessed using the AllerTOP tool [34], which is an alignment-free server for the in silico prediction of allergens based on the physicochemical properties of the amino acid sequence. As shown in Figure 3, a total of 47 peptides from myosin of *Todarodes pacificus* were predicted to be non-allergens. Next, these peptides were assessed for their potential toxicity by ToxinPred [35]. The results showed that all myosin hydrolysates were non-toxic, and no peptides with potential toxicity were predicted. Finally, the intestinal epithelial permeability of the selected peptides was evaluated using the ‘the rule of 5’, which predicted that poor absorption or permeation was more likely when there were more than five H-bond donors, ten H-bond acceptors, the molecular weight (MWT) was greater than five hundred, and the Log*P* was greater than five [36]. The results in Figure 3 indicated that only 30 ACE inhibitory peptides remained after the final permeability screening, of which 21 peptides were known antihypertensive peptides (Ala-Phe, Ala-Gly, Ala-His, Ala-Ser-Leu, Ala-Tyr, Asp-Tyr, Ile-Phe, Ile-Leu, Ile-Arg, Pro-Gly, Pro-His, Pro-Pro-Lys, Gln-Lys, Ser-Phe, Ser-Gly, Ser-Thr, Thr-Phe, Thr-Pro, Val-Ala-Phe, Val-Phe, and Val-Pro) and the remaining nine peptides were new peptides (Asn-Ala-Ile-Pro, Ala-Lys, Ile-Ile-Tyr, Asn-His, Asn-Pro-Met, Asn-Pro-Pro-Lys, Gln-Met, Gln-Tyr, and Ser-Ile). Finally, the predicted physicochemical properties of all the peptides are summarized in Tables S5 and S6, and the location of nine new ACE inhibitory peptides in the sequence of the myosin heavy chain of *Todarodes pacificus* was plotted in Figure S1.

### 2.4. ACE Inhibitory Activity of Peptides Derived from Todarodes Pacificus

**Figure 4:**
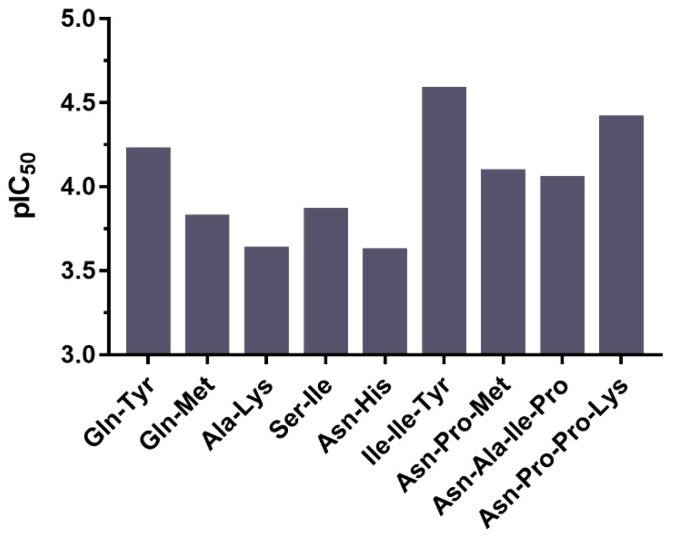
pIC_50_ values of nine new ACE inhibitory peptides from *Todarodes pacificus*.

As mentioned above, this study explored 21 known and 9 new ACE inhibitory peptides from the myosin heavy chain of *Todarodes pacificus*, which were non-toxic, non-allergic, and had good gastrointestinal stability and intestinal permeability. Among the known peptides, the one with the strongest ACE inhibitory capacity was the Ser-Thr (pIC_50_ = 5.39). This dipeptide was also found in marine shrimp *Acetes chinensis* fermented by *Lactobacillus fermentum* SM 605 [37]. Another dipeptide, Ala-Tyr, was observed to have a relatively good inhibitory effect on ACE (pIC_50_ = 4.85), which was also identified in the case of maize hydrolyzed using alcalase [38]. Moreover, in order to evaluate the ACE inhibitory ability of the newly discovered peptides, they were initially synthesized by a solid phase procedure and purified using a semi-preparative reversed-phase column. Then, their inhibitory rates were determined by an in vitro method, and the pIC_50_ values were calculated in Figure 4. The results indicated the tripeptide, Ile-Ile-Tyr, possessed the maximum pIC_50_ value of 4.58, and a sequence similar thereto has not been reported so far. The peptide with a second-best inhibitory effect on ACE was Asn-Pro-Pro-Lys (pIC_50_ = 4.41). The sequence of this tetrapeptide was reported to be similar to the ACE inhibitor from porcine skeletal muscle hydrolysate, namely Met-Asn-Pro-Pro-Lys [39], however its inhibition of ACE (pIC_50_ = 3.02) was significantly lower than Asn-Pro-Pro-Lys. These two new peptides had a great antihypertensive potential and their interaction with ACE needed a further study. 

### 2.5. Inhibition Mechanisms of ACE by Ile-Ile-Tyr and Asn-Pro-Pro-Lys

As discussed above, this study explored the in vitro ACE inhibitory activity of nine new peptides, indicating that Ile-Ile-Tyr and Asn-Pro-Pro-Lys from the myosin heavy chain of *Todarodes pacificus* had antihypertensive potential. Thus, their interaction mechanism and bonding configurations with ACE was further studied using molecular docking and MD simulation.

#### 2.5.1. Molecular Docking 

**Figure 5:**
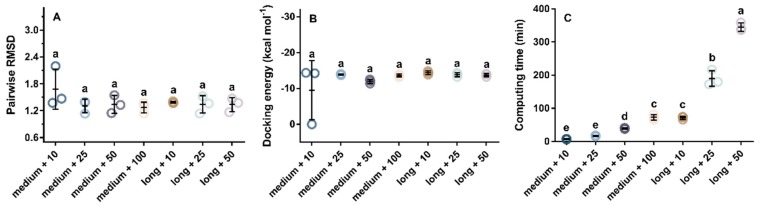
Effect of the maximum number of energy evaluations (medium and long) and the number of genetic algorithms (10, 25, 50, and 100) on the docking result of lisinopril with ACE: pairwise RMSD (**A**), docking energy (**B**), and computing time (**C**). RMSD was root mean square deviation.

The molecular docking could be used to predict the preferred orientation of these two peptides when they were bound to ACE to form a complex. Prior to the experiments, the docking parameters, including the maximum number of energy evaluations and the number of genetic algorithms, were studied and selected to better investigate the docking between ACE and its inhibitory peptide. The crystal structure of the human ACE in complex with lisinopril was used as the standard model [40]. The ligand, lisinopril, was extracted and re-docked to the active site of ACE. As shown in Figure 5A, in all docking experiments, the root mean square deviation (RMSD) difference between the docked lisinopril and the crystalline lisinopril was not significant. However, searching for 10 conformations of the docking procedure at a medium level of energy evaluations (2.5 × 10^6^) resulted in poor precision with a relative standard deviation (RSD) of 26.8%. Similarly, the docking energy in Figure 5B indicated that the docking precision was also poor at a medium level of energy evaluations and 10 structural searches. The docking was repeated three times, one of which did not achieve an effective docking energy. For computational time consumption, the data in Figure 5C indicated that docking at a long level of energy assessment (2.5 × 10^7^), or searching for 100 candidate structures, would take more than an hour to complete in our computer. At the medium level (2.5 × 10^6^), the docking experiment examining 25 alternative conformations could be completed in 20 min. Moreover, the RSD of RMSD and docking energy under these conditions were small. Therefore, the maximum number of energy evaluations and the number of genetic algorithms were set to 2.5 × 10^6^ and 25, respectively.

**Figure 6:**
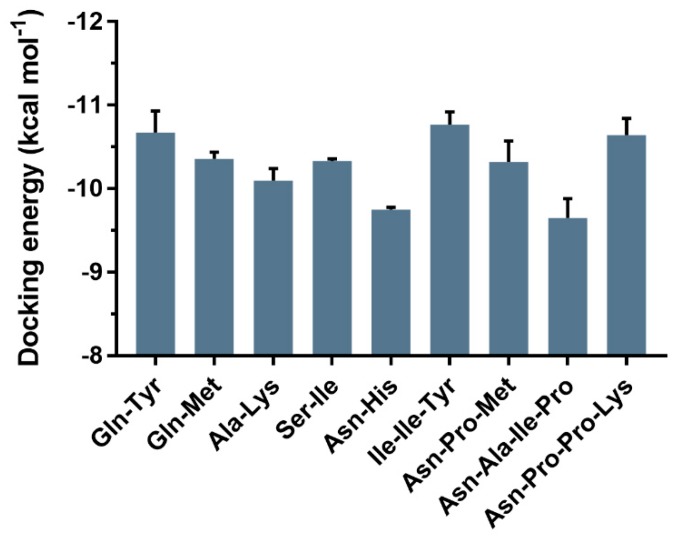
Docking energy of nine new ACE inhibitory peptides from *Todarodes pacificus*. Docking was performed according to the following parameters: maximum number of generations of 2.7 × 10^4^, rate of gene mutation of 0.02, rate of crossover of 0.8, maximum number of energy evaluations of 2.5 × 10^6^, number of genetic algorithms of 25.

Then, using the selected parameters, the docking of ACE with new inhibitory peptides was carried out. The data in Figure 6 showed that the docking energies of the test peptides were consistent with their pIC_50_ to some extent. Among them, Ile-Ile-Tyr and Asn-Pro-Pro-Lys had lower docking energy with values of −10.87 and −10.74 kcal·mol^−1^, respectively, which were also in line with their respective pIC_50_. 

**Figure 7:**
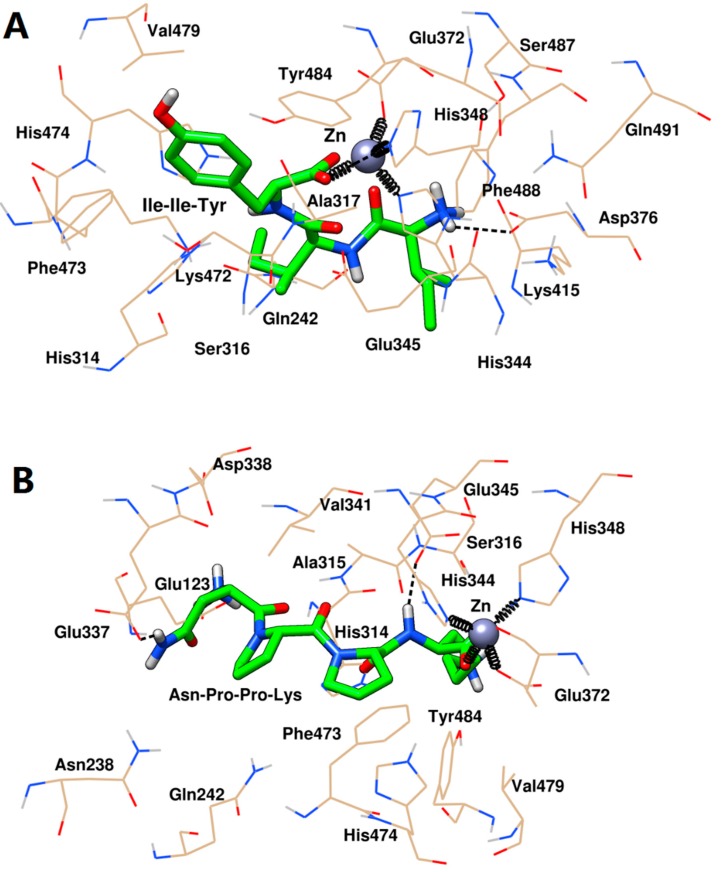
Docking conformation of Ile-Ile-Tyr (**A**) and Asn-Pro-Pro-Lys (**B**) with ACE. Gold lines represented the ACE residues involved in interacting with peptides. Green sticks represented the peptides. Grey sphere represented the Zn(II). Black dash lines represented the hydrogen bonds. Black springs represented the coordination bonds.

The docking conformation in Figure 7 revealed that Ile-Ile-Tyr and Asn-Pro-Pro-Lys could be embedded in the S_1_ hydrophobic pocket of ACE (Gln242, His344, His348, Glu372, Tyr484, and Glu345). The Asn-Pro-Pro-Lys was also close to Ala315 in the S_1_ pocket, while Asn-Pro-Pro-Lys did not interact with this residue. For the S_2_ pocket, Ile-Ile-Tyr was bound tightly to His314, Lys472, and His474 at this site, while Asn-Pro-Pro-Lys was only located near His314 and His474. In addition, Asn-Pro-Pro-Lys could interact with the Glu123 residue in the S_1_’ pocket. Further, analysis of the interaction between these two inhibitors and the surrounding amino acid residues at the active site of ACE indicated that there were three molecular forces involved in the stabilization of the peptides, including coordination, hydrogen bonding, and hydrophobic interaction. Figure 7 showed that Ile-Ile-Tyr and Asn-Pro-Pro-Lys could coordinate with the active site Zn(II) of ACE. The oxygen atom at the C-terminus of the peptide was in contact with Glu372, His344, and His348 by coordination interaction. As shown in Figure 7A, the Ile-Ile-Tyr formed two hydrogen bonds by reacting with the His348 and Asp346 residues of ACE. This tripeptide also entered the hydrophobic pocket of ACE and formed hydrophobic interactions with the Gln242, His314, Ser316, Ala317, Glu345, Glu372, Lys415, Lys472, Phe473, His474, Val479, Ser487, Phe488, and Gln491 residues. As shown in Figure 7B, the docking result of Asn-Pro-Pro-Lys and ACE was similar to those of the previous Ile-Ile-Tyr, forming two hydrogen bonds with the Glu337 and Glu345 residues. This tetrapeptide also interacted hydrophobically with the residues of Glu123, Asn238, Gln242, His314, Ala315, Ser316, Asp338, Val341, Phe473, His474, Val479, and Tyr484.

#### 2.5.2. MD Simulation 

Based on the docking conformation, MD simulation based on Ambertools with the metal center parameter builder (MCPB) was used to study the stable state of the complex of ACE with Ile-Ile-Tyr and Asn-Pro-Pro-Lys, and to further explore the interaction mechanism. 

**Figure 8:**
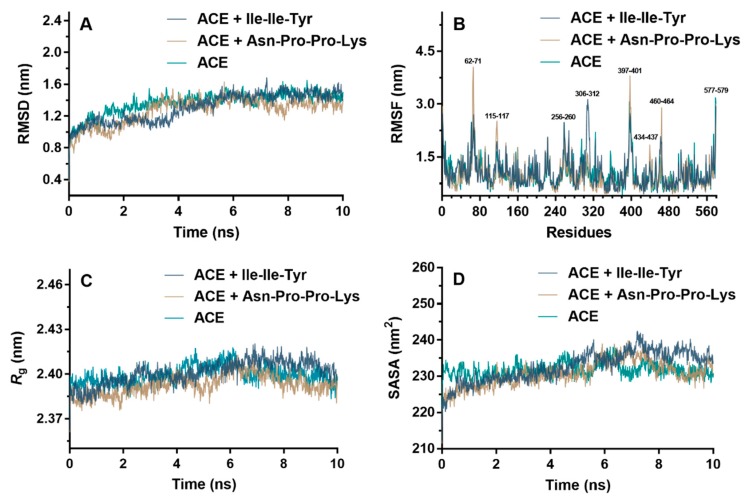
Molecular dynamics results of ACE with and without peptides Ile-Ile-Tyr and Asn-Pro-Pro-Lys: RMSD (**A**), RMSF (**B**), *R*_g_ (**C**), SASA (**D**). RMSD was root mean square deviation. RMSF was root mean square fluctuation. *R*_g_ was radius of gyration, SASA was solvent accessible surface area.

The variations of the RMSD regarding ACE with or without peptides are shown in Figure 8A. In general, if the RMSD value of a typical dynamic box fluctuated within 0.1 nm, the system could be considered to have reached a balanced steady state [41]. Obviously, the pure ACE and two ACE-peptide complexes were stable in 10 ns. Similar to RMSD, the root mean square fluctuation (RMSF) was used to analyze the difference in atomic position between a conformation of a biological macromolecule at a certain time and a reference conformation. The larger the RMSF of a residue of a protein, the higher its flexibility [42]. As shown in Figure 8B, ACE had five major flexible regions located at the 62–71, 115–117, 256–260, 306–312, 397–401, 434–437, 460–464, 503–506, and 577–579 residues, which were the peripheral random coil structures, away from the active center (Figure S2). After binding Ile-Ile-Tyr and Asn-Pro-Pro-Lys, ACE generally maintained a more stable conformation. The radius of gyration (*R*_g_) was used to describe the tightness of the protein structure. The greater the value, the higher the degree of protein unfolding [43]. The data in Figure 8C indicated that the average *R*_g_ of the ACE−peptide complex system was indistinguishable from the receptor ACE itself, indicating that the tightness of ACE did not change significantly after binding Ile-Ile-Tyr and Asn-Pro-Pro-Lys, and remained stable. To further realize the variation of *R*_g_, the solvent accessible surface area (SASA) of free and peptide-binding ACE were studied. The SASA was defined as the surface area of the proteins that interacted with its solvent molecules, which had a certain correlation with the surface hydrophobicity of proteins [44]. The data in Figure 8D showed that the SASA of the system containing peptides was not significantly different from the pure ACE, indicating that the surface area of the ACE in contact with the solvent remained substantially the same before and after the complex formation with Ile-Ile-Tyr and Asn-Pro-Pro-Lys. This result also suggested the structure of ACE was stable after interaction with these two inhibitory peptides.

**Figure 9:**
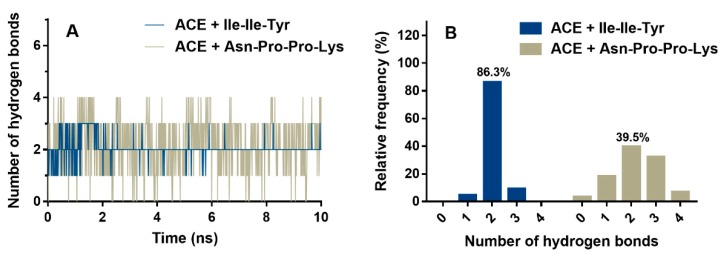
Number of hydrogen bonds between ACE and inhibitory peptides during molecular dynamics (**A**) and its frequency histogram (**B**).

Figure 9A shows the hydrogen-bonding interactions between ACE and inhibitory peptides during the MD process. Throughout the simulation process, Asn-Pro-Pro-Lys can form more than three hydrogen bonds with ACE. However, for Ile-Ile-Tyr, the number of hydrogen bonds in the entire MD process can rarely exceed three. As shown in Figure 9B, the models of two hydrogen bonds were the stable conformations of ACE with Ile-Ile-Tyr and Asn-Pro-Pro-Lys, with frequencies of 86.3% and 39.5%, respectively. Additionally, in order to evaluate the coordination during MD, the distances between Zn(II) and its surrounding residues were calculated, including the His344, His348, Glu372 of ACE, and C-terminal residues (Tyr or Lys) of the inhibitory peptides. 

**Figure 10:**
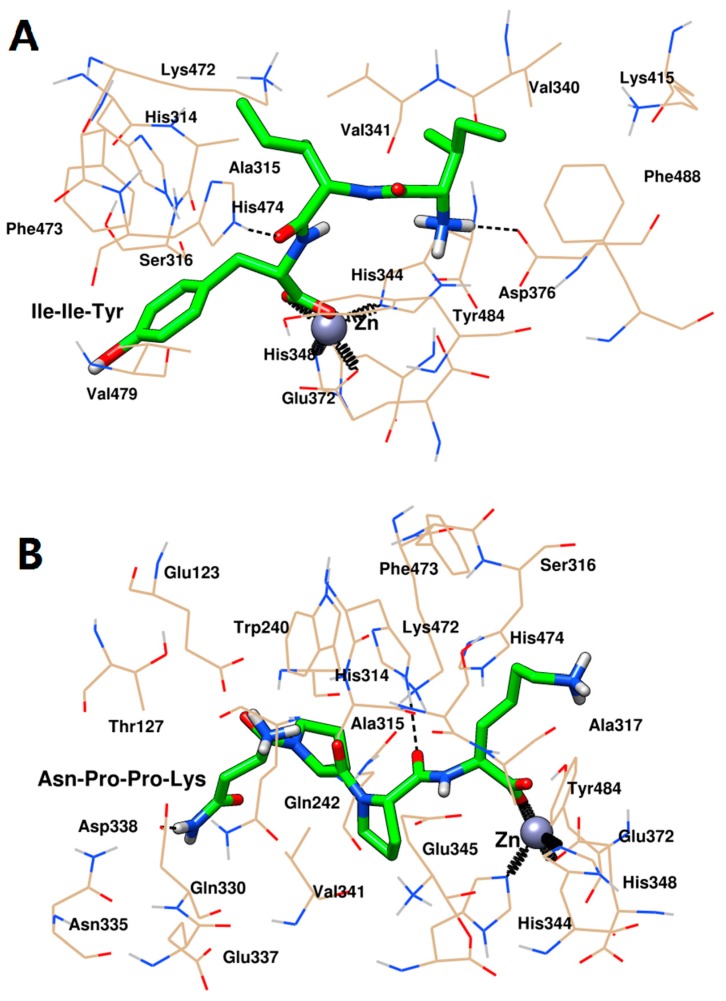
Molecular dynamics conformation of Ile-Ile-Tyr (**A**) and Asn-Pro-Pro-Lys (**B**) with ACE. Gold lines represented the ACE residues involved in interacting with peptides. Green sticks represented the peptides. Grey sphere represented the Zn(II). Black dash lines represented the hydrogen bonds. Black springs represented the coordination bonds.

Finally, the interaction between the two peptides and the active site of the target was further revealed by selecting the stable conformations of ACE with Ile-Ile-Tyr and Asn-Pro-Pro-Lys after the end of MD. As shown in Figure 10A, the His344, His348, and Glu372 residues in ACE and Ile-Ile-Tyr together formed coordinate bonds with Zn(II). Two hydrogen bonds were formed between the His474 and Asp376 residues in ACE and Ile-Ile-Tyr. Other amino acid residues, such as the His314, Ala315, Ser316, Val340, Val341, Lys415, Lys472, Phe473, Val479, Tyr484, and Phe488 residues, constituted a hydrophobic pocket surrounding the Ile-Ile-Tyr. As shown in Figure 10B, similarly, there was a coordination among the Asn-Pro-Pro-Lys, Zn(II), His344, His348, and Glu372 residues. Two hydrogen bonds were formed with the participation of the His314 and Asp388 residues. The other residues in ACE, including Glu123, Thr127, Trp240, Gln242, Ala315, Ser316, Ala317, Gln330, Asn335, Glu337, Glu345, Val341, Lys472, Phe473, His474, and Tyr484, could stabilize Asn-Pro-Pro-Lys by hydrophobic interactions. Moreover, these two peptides were observed to bind to the S_1_ pocket of ACE (Ala315, His344, His348, Glu372, and Tyr484) upon forming the stable complexes. Similar to Figure 7, Asn-Pro-Pro-Lys could also interact with Glu345 in the S_1_ pocket, while Ile-Ile-Tyr did not. After MD simulation, they were able to approach His314, Lys472, and His474 in the S_2_ pocket, suggesting a tighter bonding configuration. Besides, Asn-Pro-Pro-Lys could also be close to Gln242 by hydrophobic force, which was another residue in the S_2_ pocket. For the S_1_’ pocket, Ile-Ile-Tyr was alienated from it, but there was still an interaction between Asn-Pro-Pro-Lys and the Glu123 residue at this site.

## 3. Materials and Methods

### 3.1. Reagents

The tested peptides from t hmyosin heavy chain in *Todarodes pacificus* were provided by Anhui Guoping Pharmaceutical Co., Ltd. (Hefei, China). The ACE from rabbit lungs, hippuryl-histidyl-leucine (HHL), and hippuric acid (HA) were purchased from the Sigma Chemical Company (St. Louis, MO, USA). The solvents for high performance liquid chromatography (HPLC) including acetonitrile and trifluoroacetic acid (TFA) were provided by Merck (Darmstadt, Germany). All the other reagents and chemicals used were of analytical grade.

### 3.2. In Silico Hydrolysis

The myosin heavy chain (GenBank: ADU19853.1) from *Todarodes pacificus* was obtained from the NCBI protein database, available at https://www.ncbi.nlm.nih.gov/protein/. Then, it was hydrolyzed independently by the papain (EC 3.4.22.2), ficin (EC 3.4.22.3), and a combination of them using the BIOPEP-UWM database [27], available at http://www.uwm.edu.pl/biochemia/index.php/en/biopep. After hydrolysis by the plant proteases, the PREP (EC 3.4.21.26) was introduced and combined with the above three groups of proteases for supplemental digestion to produce a peptide with a C-terminal Pro residue. When performing this process, it was worth noting that the PREP only cleaved substrates whose sequences did not exceed 30 amino acids. Furthermore, when the Pro-Pro sequence was present at the C-terminus, its activity was blocked [45].

### 3.3. Prediction of ACE Inhibitory Peptides

The search of di- and tripeptides from myosin was performed using the database BIOPEP-UWM. Moreover, the *DH*, *A*_E_, and *W* of release of fragments with a given type of activity by selected enzymes were also were calculated [27]. Furthermore, the tetra-, penta-, and hexapeptides with ACE inhibitory activity were predicted via the AHTpin database, available at http://crdd.osdd.net/raghava/ahtpin/index.php, which used support vector machine (SVM) regression and classification models based on amino acid composition prediction [30]. In this work, it was trained at a threshold of 0.0, i.e., any peptide predicted over a 0.0 threshold was labeled as bioactive.

### 3.4. In Silico Prediction of Physicochemical Properties of Peptides

Additional in silico analysis was performed to predict the toxicity, allergenicity, gastrointestinal stability, and intestinal epithelial permeability. For prediction of the toxic/non-toxic peptides, the ToxinPred tool was used based on the SVM module [35], available at http://crdd.osdd.net/raghava/toxinpred/. The SVM threshold was selected as 0.0. The allergenicity was predicted using the AllerTOP tool [34], available at http://www.ddg-pharmfac.net/AllerTOP/. This method was based on auto cross covariance (ACC) transformation of protein sequences into uniform equal-length vectors. The principal properties of the amino acids were represented by five E descriptors and the proteins are classified by the k-nearest neighbor algorithm (kNN, k = 1) based on a training set containing 2427 known allergens from different species and 2427 non-allergens. The resistance to digestion was predicted using pepsin (EC 3.4.21.1), trypsin (EC 3.4.21.4), and chymotrypsin (EC 3.4.21.2) in the ExPASy PeptideCutter database, available at https://web.expasy.org/peptide_cutter/. Finally, the intestinal epithelial permeability was assessed by ‘the rule of 5’, involving H-bond donors, H-bond acceptors, MWT, and Log*P* [36]. These parameters were calculated by XLOGP3 [46], available at http://www.sioc-ccbg.ac.cn/?p=42&software=xlogp3.

### 3.5. In Vitro ACE Inhibitory Activity 

The ACE inhibitory activity analysis was performed based on Yu et al. with some modifications [16]. HHL was dissolved in 100 mM sodium borate buffer (pH 8.3) to achieve a final concentration of 5.8 mM. A reaction mixture containing 50 μL of ACE solution (100 mU/mL) and 100 μL of sample were pre-incubated at 37 °C for 5 min. Subsequently, 150 μL HHL was added and the mixture was incubated at 37 °C for 60 min. The reaction was terminated by the addition of 200 μL of 0.1 M HCl. The released HA was extracted by the addition of 1.0 mL ethyl acetate followed by centrifugation at 1000× *g* and 4 °C for 15 min. The upper layer was transferred into a new tube and purged with a stream of nitrogen. Then, 1 mL of deionized water was added. The amount of HA was determined via an ultra-performance liquid chromatography on a Waters Acquity UPLC BEH C18 column (1.7 μm, 2.1 mm × 100 mm). The dissolved samples were filtered through 0.22-μm syringe filters, and 10 μL were injected. The column was eluted with mobile phase (A) of 0.1% TFA in water and (B) of 25% acetonitrile at flow rates of 0.2 mL/min. The elution was observed at 228 nm. A series of standard HA solutions were prepared to construct a calibration curve. The degree of ACE inhibition was calculated as follows:

(1) $$\mathrm{ACE}\;\mathrm{inhibition}\;\mathrm{activity}\;\left(\%\right)=\frac{A-B}{A}\times 100\%$$

where *A* was the HA concentration of a reaction containing ACE without peptide, B was the HA concentration of a reaction in the presence of ACE and peptide. The IC_50_ was defined as the concentration of inhibitor required to inhibit 50% of the ACE activity.

### 3.6. Molecular Docking of ACE with Peptides

#### 3.6.1. Selection of Docking Parameters 

The AutoDock 4.2 package was used for the docking simulation based on a Lamarckian genetic algorithm [47]. Before the docking of our selected peptides with ACE, the conformation search parameters were optimized. Lisinopril from the crystal file (PDB 1O86) was selected as the experimental ligand. It was converted to a PDBQT file for later docking after merging non-polar hydrogens, assigning the polar hydrogens, and selecting the rotatable bonds. The zinc-centered map for ACE was calculated using an AutoGrid with 50 × 70 × 50 grid points of 0.375 Å spacing. The maximum number of generations was set to 2.7 × 10^4^. The rate of gene mutation was set to 0.02. The rate of crossover was set to 0.8. The maximum number of energy evaluations was evaluated at the medium level (2.5 × 10^6^) and the long level (2.5 × 10^7^), and the number of genetic algorithms was examined at 10, 25, 50, and 100. The docking result was mainly evaluated by the RMSD difference between the docking structure and the structure of lisinopril in the crystal ACE (PDB 1O86). Other indexes, such as the computing time and docking energy, were also considered.

#### 3.6.2. Docking of ACE with Inhibitory Peptides

The models of Ile-Ile-Tyr and Asn-Pro-Pro-Lys for docking were generated by the Chimera program [48]. AutoDockTools 1.5.6rc3 was utilized to create the PDBQT format files by merging non-polar hydrogens, assigning the polar hydrogens, and selecting the rotatable bonds [47]. The AutoDock 4.2 program was used to determine the possible ACE–peptide conformation [47]. The processing parameters are those selected in Section 3.6.1. Finally, the Chimera package was used for the visualization of molecular docking results [48].

### 3.7. MD of ACE with Inhibitory Peptides

**Figure 11:**
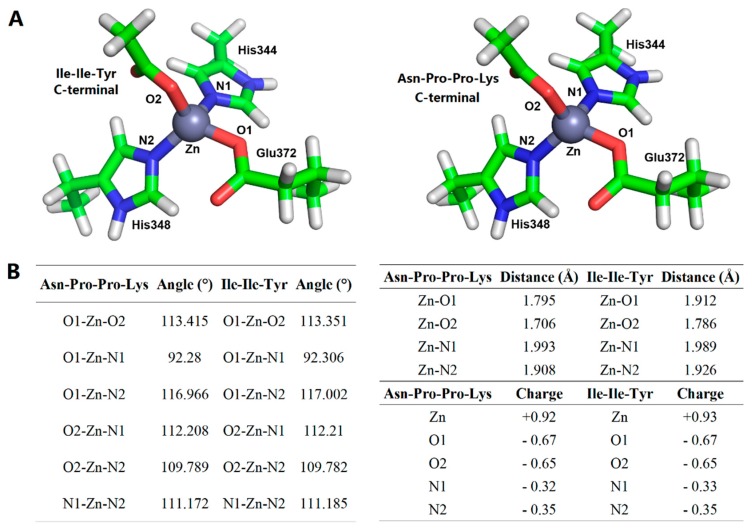
Coordination conformation (**A**) and related parameters (**B**) between ACE active center Zn(II) and inhibitory peptides (Ile-Ile-Tyr and Asn-Pro-Pro-Lys).

The initial conformations of ACE with peptides were obtained based on Section 3.6.2 and then simulated by all-atom MD using the AmberTools 18 package [49]. The Amber ff14SB all-atom force field was applied to simulate the complex of ACE with its peptides [50], which were solvated in a cubical periodic box with a 1.0 nm solute-wall distance using the transferable interatomic potential with three points model (TIP3P) water model. The coordinate bonds between Zn(II) and its surroundings were treated by the MCPB method [51], and the parameters of the amino acids coordinated with Zn(II) were obtained at the B3LYP/6-31G* level (Figure 11). Two phases of minimization were performed for the initial structures before the MD simulations. First, the solute atoms were restrained, and water molecules were minimized for 5000 steps with the steepest descent method followed by 5000 steps of conjugate gradient. Second, the unrestrained minimizations containing 5000 steps steepest descent and 5000 steps conjugate gradient of the whole systems were executed. The MD simulations were also set to two stages. First, the solutes were restrained, and the systems were slowly heated from 0 to 300 K over 50 ps. Then, non-restraint MD simulations at 300 K were performed for 10 ns. In these simulations, the SHAKE algorithm was applied to constrain covalent bonds. The MD time step was taken as 1 fs, and one snapshot was sampled every 10,000 steps (10 ps), thus 1000 conformations were obtained in each MD simulation. The Chimera package was used for the visualization of the MD results [48].

## 4. Conclusions

In this work, nine novel ACE inhibitory peptides with short amino acids sequences were predicted and screened from *Todarodes pacificus* by an integrated bioinformatics approach. Among them, the tripeptide Ile-Ile-Tyr and tetrapeptide Asn-Pro-Pro-Lys exhibited strong inhibitory effects on ACE with pIC_50_ values of 4.58 and 4.41, respectively, which were also in line with their docking energies. Furthermore, MD simulation with the MCPB method showed that these two peptides could be deeply buried in the hydrophobic pockets of S_1_ and S_2_ of ACE, which were stabilized by coordination bonds with zinc ions and hydrogen bonding and hydrophobic interactions with surrounding residues. Thus, the present study demonstrated the potential of *Todarodes pacificus* as a suitable raw material for the industrial production of ACE-inhibitory peptides, which could be used to effectively treat hypertension. 

## Supplementary Materials

Supplementary materials can be found at https://www.mdpi.com/1422-0067/20/17/4159/s1.
