# Supplementary material for: Discovery of Novel Angiotensin-Converting Enzyme Inhibitory Peptides from Todarodes pacificus and Their Inhibitory Mechanism: In Silico and In Vitro Studies
Source: Int J Mol Sci. 2019 Aug 26;20(17):4159. doi: 10.3390/ijms20174159 (PMC6747323; doi:10.3390/ijms20174159)

Supplementary Materials

Table S1 ACE inhibitory di- and tripeptides released from the myosin heavy chain of *Todarodes pacificus* hydrolyzed by papain, ficin and papain−ficin *in silico*

Peptide	pIC_{50}[a]	Source[b]	Peptide	pIC_{50}[a]	Source[b]
AF	3.78	Soybean	NF	4.33	Garlic (*Allium sativum* L)
AG	2.60	Carp parvalbumin	NG	1.92	Chicken (*Gallus gallus*)
AH	3.28	Sweet potato tuber	NK	3.09	Pork sarcoplasmic proteins
AR	4.02	Pork skeletal muscle	NY	4.49	Garlic (*Allium sativum* L)
AY	4.85	Soybean	PG	1.77	Pork sarcoplasmic proteins
DG	5.67	Soybean	PH	/	Pork sarcoplasmic proteins
DY	4.00	Oyster protein hydrolysate	PL	3.47	Pork sarcoplasmic proteins
EG	2.00	Pork sarcoplasmic proteins	QG	2.13	Carp parvalbumin
EI	/	Chicken (*Gallus gallus*)	QK	3.05	Pork sarcoplasmic proteins
EK	/	Chicken (*Gallus gallus*)	SF	3.89	Garlic (*Allium sativum L*)
EW	3.86	Chicken (*Gallus gallus*)	SG	2.07	Carp parvalbumin
EY	5.57	Shark meat	ST	5.39	Marine shrimp (*Acetes chinensis*)
HL	2.49	Pork sarcoplasmic proteins	TF	4.74	Rapeseed proteins
IF	4.18	Soybean sauce	VE	/	Pork sarcoplasmic proteins
IG	2.92	Carp parvalbumin	VF	5.04	Wakame (*Undaria pinnatifida*)
IL	4.26	/	VG	2.96	Soybean
IR	3.16	Bovine β-lactoglobulin	VK	4.89	Carp parvalbumin
IY	5.68	Dried bonito	VR	4.28	Ovine milk proteins
KF	4.55	Wakame (*Undaria pinnatifida*)	WL	4.52	Soybean
KG	2.49	Pork sarcoplasmic proteins	YG	2.82	Pork sarcoplasmic proteins
KL	4.30	Carp parvalbumin	YL	4.09	Sardine muscle (*Sardina pilchardus*)
KR	3.42	Pork sarcoplasmic proteins	AEL	4.24	Algae (*Chlorella vulgaris*)
MF	6.04	Shark	ASL	3.99	Silkworm pupa (*Bombyx mori*)
MG	2.32	Chicken (*Gallus gallus*)	NKL	4.06	Bovine β-casein
MM	3.26	Poultry protein	PPK	3.00	Porcine skeletal muscle protein
MY	3.71	Carp parvalbumin	VAF	4.45	Algae (*Spirulina platensis*)

[a] pIC_{50} is derived from the AHTpin and BIOPEP-UWM databases

[b] Sources of peptides are provided by the BIOPEP-UWM and AHTPDB databases

Table S2 ACE inhibitory di- and tripeptides released from the myosin heavy chain of *Todarodes pacificus* hydrolyzed by papain−prolyl endopeptidase, ficin−prolyl endopeptidase, and papain−ficin−prolyl endopeptidase *in silico*

Peptide	pIC_{50}[a]	Source[b]	Peptide	pIC_{50}[a]	Source[b]
AF	3.78	Soybean	MY	3.71	Sardine muscle (*Sardina pilchardus*)
AG	2.60	Carp parvalbumin	NF	4.33	Garlic (*Allium sativum L*)
AH	3.28	Sweet potato tuber	NG	1.92	Chicken (*Gallus gallus*)
AP	4.54	Atlantic salmon	NK	3.09	Pork sarcoplasmic proteins
AR	4.02	Algae (*Chlorella vulgaris*)	QG	2.13	Carp parvalbumin
AY	4.85	Soybean	QK	3.05	Pork sarcoplasmic proteins
DG	5.67	Soybean	SF	3.89	Garlic (*Allium sativum L*)
DY	4.00	Oyster protein hydrolysate	SG	2.07	Carp parvalbumin
EG	2.00	Pork sarcoplasmic proteins	ST	5.39	Marine shrimp (*Acetes chinensis*)
EI	/	Chicken (*Gallus gallus*)	TF	4.74	Rapeseed proteins
EK	/	Chicken (*Gallus gallus*)	TP	3.54	Anchovy sauce
HL	2.49	Pork sarcoplasmic proteins	VF	5.04	Wakame (*Undaria pinnatifida*)
IF	4.18	Soybean sauce	VG	2.96	Soybean
IG	2.92	Carp parvalbumin	VK	4.89	Carp parvalbumin
IL	4.26	/	VP	3.38	Soybean
IR	3.16	Bovine β-lactoglobulin	VR	4.78	Ovine milk proteins
IY	5.68	Dried bonito	YG	2.82	Pork sarcoplasmic proteins
KF	4.55	Wakame (*Undaria pinnatifida*)	YL	4.09	Carp parvalbumin
KG	2.49	Pork sarcoplasmic proteins	AEL	4.24	Algae (*Chlorella vulgaris*)
KL	4.30	Carp parvalbumin	AIP	3.46	Bovine β-lactoglobulin
KP	4.66	Anchovy sauce	ASL	3.99	Silkworm pupa (*Bombyx mori*)
KR	3.42	Pork sarcoplasmic proteins	AVP	3.47	Bovine β-casein
MF	6.04	Shark	NKL	4.06	Bovine β-casein
MG	2.32	Chicken (*Gallus gallus*)	VAF	4.45	Algae (*Spirulina platensis*)
MM	3.26	Poultry protein			

[a] pIC_{50} is derived from the AHTpin and BIOPEP-UWM databases

[b] Sources of peptides are provided by the BIOPEP-UWM and AHTPDB databases

Table S3 ACE inhibitory tetra-, penta-, and hexapeptides released from the myosin heavy chain of *Todarodes pacificus* hydrolyzed by papain, ficin and papain−ficin *in silico*

Peptide	SVM score[a]	Peptide	SVM score[a]	Peptide	SVM score[a]
ADIKEL	0.10	EKKIR	0.36	PIYT	1.06
AEKV	0.05	EKSR	1.00	PPEPDT	0.27
AEMPPH	0.34	EMKWK	0.48	QCNPVL	0.23
AEQEK	0.37	ENAQK	0.35	QDHEN	0.30
AETQK	0.25	EPIVK	0.87	QEQDH	0.86
AINPY	0.71	EQEEY	0.38	QESKT	0.14
AINPYR	0.99	HIKEL	0.68	QKNDL	0.32
AIPS	0.57	HKESL	0.39	QKVKSR	0.16
AKEHF	0.33	IIYSEF	0.14	QMNPPK	0.23
AKNR	0.21	IKKKL	0.59	QVKEL	0.75
AVPDPG	0.37	KKKKG	0.40	SKVEK	0.19
AYKKL	0.77	KNPMF	0.20	VDKYR	0.28
CEQMR	0.46	KNYER	0.55	VIQY	0.91
CIEAH	0.52	MQQK	0.32	VQQEL	0.71
CINYT	0.56	NHHMF	0.14	YDNHL	0.34
DNQIK	0.22	NKVKPL	0.02	YESG	0.70
DQIDQL	0.19	NPMF	0.54		
EEVQK	0.65	NYHIF	0.51		

[a] SVM score above 0 means the peptide is predicted to be antihypertensive using the AHTpin database

Table S4 ACE inhibitory tetra-, penta-, and hexapeptides released from *Todarodes pacificus* myosin hydrolyzed by papain−prolyl endopeptidase, ficin−prolyl endopeptidase and papain−ficin−prolyl endopeptidase *in silico*

Peptide	SVM score[a]	Peptide	SVM score[a]	Peptide	SVM score[a]
ADIKEL	0.10	ENAQK	0.35	QDHEN	0.30
AEMPP	0.50	EQEEY	0.38	QEQDH	0.86
AEQEK	0.37	EYVT	0.05	QESKT	0.14
AETQK	0.25	HIKEL	0.68	QKNDL	0.32
AINP	0.34	IEKP	0.50	QKVKSR	0.16
AKEHF	0.33	IIYSEF	0.14	QMNPP	0.98
AYKKL	0.77	IKKKL	0.59	QVKEL	0.75
CEQMR	0.46	KIKVG	0.33	QYML	1.06
CIAINP	0.28	KKKKG	0.40	SKEP	0.32
CIEAH	0.52	KNYER	0.55	SKVEK	0.19
CIIP	0.62	MQQK	0.32	VDKYR	0.28
CINYT	0.56	NAIP	0.34	VIQY	0.91
DNQIK	0.22	NCWVP	1.30	VQQEL	0.71
DQIDQL	0.19	NHHMF	0.14	YDNHL	0.34
EEVQK	0.65	NKVKP	1.20	YSIL	0.21
EKKIR	0.36	NYHIF	0.51		
EMKWK	0.48	QCNP	0.78		

[a] SVM score above 0 means the peptide is predicted to be antihypertensive using the AHTpin database

Table S5 Predicted toxicity, allergenicity, gastrointestinal stability, and intestinal epithelial permeability of selected peptides

Peptide	Toxicity	Allergenicity	CLog*P*	H-bond donors	H-bond acceptors	MWT	Gastrointestinal stability
AF	Non	Non	−2.47	3	3	236.3	Good
AG	Non	Non	−3.65	3	3	146.1	Good
AH	Non	Non	−3.84	4	4	226.2	Good
AL	Non	Allergen	−2.78	3	3	202.3	Good
AP	Non	Allergen	−2.71	2	3	186.2	Good
AR	Non	Allergen	−4.98	5	3	245.3	Good
AY	Non	Non	−3.48	4	4	252.3	Good
DG	Non	Allergen	−4.52	4	5	190.2	Good
DY	Non	Non	−4.35	5	6	296.3	Good
EG	Non	Allergen	−2.66	4	5	204.2	Good
EI	Non	Allergen	−3.47	4	5	260.3	Good
EK	Non	Allergen	−6.28	5	5	275.3	Good
EW	Non	Allergen	−2.65	5	5	333.3	Good
EY	Non	Allergen	−3.77	5	6	310.3	Good
HL	Non	Non	−2.17	3	4	268.3	H-L
IF	Non	Non	−1.81	3	3	278.3	Good
IG	Non	Allergen	−2.50	3	3	188.2	Good
IL	Non	Non	−1.71	3	3	244.3	Good
IR	Non	Non	−3.86	5	3	287.4	Good
IY	Non	Allergen	−2.16	4	4	294.3	Good
KF	Non	Allergen	−2.71	4	3	293.4	K-F
KG	Non	Non	−3.89	4	3	203.2	K-G
KL	Non	Non	−3.02	4	3	259.3	K-L
KP	Non	Non	−3.32	3	3	243.3	K-P
KR	Non	Non	−4.31	6	3	302.4	K-R
MF	Non	Allergen	−1.67	3	3	296.4	M-F
MG	Non	Non	−3.01	3	3	206.3	M-G
MM	Non	Non	−2.55	3	3	280.4	M-M
MY	Non	Allergen	−2.03	4	4	312.4	Good
NF	Non	Allergen	−3.99	4	4	279.3	N-F
NG	Non	Allergen	−5.17	4	4	189.2	Good
NK	Non	Allergen	−5.01	5	4	260.3	Good
NY	Non	Allergen	−5.00	5	5	295.3	Good
PG	Non	Non	−3.81	3	3	172.2	Good
PH	Non	Non	−2.80	4	5	349.4	Good
PL	Non	Allergen	−2.41	3	3	228.3	Good
QG	Non	Allergen	−4.52	4	4	203.2	Good
QK	Non	Non	−4.65	5	4	274.3	Good
SF	Non	Non	−3.52	4	4	252.3	Good

SG	Non	Non	−4.70	4	4	162.1	Good
ST	Non	Non	−4.92	5	5	206.2	Good
TF	Non	Non	−3.09	4	4	266.3	Good
TP	Non	Non	−3.33	3	4	216.2	Good
VE	Non	Allergen	−3.90	4	5	246.3	Good
VF	Non	Non	−2.16	3	3	264.3	Good
VG	Non	Allergen	−2.69	3	3	174.2	Good
VK	Non	Allergen	−3.63	4	3	245.3	Good
VP	Non	Non	−2.15	2	3	214.3	Good
VR	Non	Allergen	−4.21	5	3	273.3	Good
WL	Non	Non	−0.73	4	3	317.4	W-L
YG	Non	Allergen	−2.86	4	4	238.2	Y-G
YL	Non	Allergen	−1.75	4	4	294.3	Y-L
AEL	Non	Allergen	−3.86	5	6	331.4	Good
AIP	Non	Allergen	−2.04	3	4	299.4	Good
ASL	Non	Non	−3.67	5	5	289.3	Good
AVP	Non	Allergen	−3.26	3	4	285.3	Good
NKL	Non	Non	−3.92	6	5	373.4	NK-L
PPK	Non	Non	−2.80	4	4	340.4	Good
VAF	Non	Non	−1.66	4	4	335.4	Good
AEKV	Non	Non	−7.41	7	7	445.5	AE-K-V
AINP	Non	Allergen	−5.34	5	6	413.5	Good
AIPS	Non	Allergen	−4.89	5	6	386.4	Good
AKNR	Non	Non	−6.33	9	6	487.6	AK-NR
CIIP	Non	Allergen	−2.58	4	5	444.6	Good
EKSR	Non	Non	−8.66	10	8	518.6	EK-SR
EYVT	Non	Non	−3.51	8	9	510.5	EY-VT
IEKP	Non	Non	−6.85	6	7	485.6	Good
MQQK	Non	Allergen	−5.68	8	7	533.6	M-QQK
NAIP	Non	Non	−5.34	5	6	413.5	Good
NPMF	Non	Allergen	−4.17	5	6	507.6	NPM-F
PIYT	Non	Non	−0.51	6	6	504.6	PIY-T
QCNP	Non	Non	−7.90	6	7	460.5	Good
QYML	Non	Non	−3.44	7	7	553.7	QY-M-L
SKEP	Non	Allergen	−9.22	7	8	459.5	SK-EP
VIQY	Non	Non	−3.57	7	7	521.6	Good
YESG	Non	Non	−5.7	8	9	454.4	Y -ESG
YSIL	Non	Non	−2.14	7	7	494.6	Y-SI-L
AEMPP	Non	Allergen	−4.96	5	8	543.6	Good
AEQEK	Non	Non	−8.71	10	11	603.6	Good
AINPY	Non	Allergen	−3.02	7	8	576.6	Good
AKEHF	Non	Allergen	−5.69	9	9	630.7	AK-EH-F
ASKQK	Non	Non	−6.70	10	8	560.6	ASK-QK
AYKKL	Non	Non	−3.64	9	7	621.8	AY-K-K-L

CEQMR	Non	Non	−5.95	10	9	665.8	CEQ-M-R
CIEAH	Non	Allergen	−3.52	8	9	571.6	Good
CINYT	Non	Non	−4.41	9	9	612.7	CINY-T
DNQIK	Non	Allergen	−8.76	10	10	616.7	DNQI-K
EEVQK	Non	Allergen	−7.75	10	10	631.7	Good
EKKIR	Non	Non	−6.78	11	8	672.8	EK-K-IR
EMKWK	Non	Allergen	−5.52	10	8	720.9	EM-K-W-K
ENAQK	Non	Allergen	−9.72	10	10	588.6	Good
EPIVK	Non	Allergen	−6.16	7	8	584.7	Good
EQEEY	Non	Non	−7.19	11	14	696.7	Good
HIKEL	Non	Non	−4.63	9	9	638.8	H-IK-E-L
HKESL	Non	Allergen	−7.00	10	10	612.7	H-K-ESL
IKKKL	Non	Non	−2.44	9	6	628.8	IK-K-K-K-L
KIKVG	Non	Non	−3.31	8	6	543.7	K-IK-VG
KKKKG	Non	Non	−5.36	10	6	587.8	K-K-K-K-G
KNPMF	Non	Non	−3.23	7	7	635.8	K-NPM-F
KNYER	Non	Non	−8.15	12	10	708.8	K-NY-ET
NCWVP	Non	Non	−2.37	7	7	617.7	NCW-VP
NHHMF	Non	Non	−3.77	9	9	684.8	NH-H-M-F
NKVKP	Non	Allergen	−6.42	8	7	584.7	NK-VKP
NYHIF	Non	Non	−1.62	9	9	692.8	NY-H-I-F
PPEPDT	Non	Allergen	−5.07	8	11	557.6	Good
QDHEN	Non	Non	−8.31	11	13	641.6	QDH-EN
QEQDH	Non	Allergen	−7.95	11	13	655.6	Good
QESKT	Non	Allergen	−9.87	10	10	591.6	QESK-T
QKNDL	Non	Allergen	−8.76	10	10	616.7	QK-NDL
QMNPP	Non	Allergen	−5.44	6	8	585.7	QM-NPP
QVKEL	Non	Allergen	−7.50	9	9	615.7	QVK-E-L
SKVEK	Non	Non	−7.36	10	9	589.7	SK-VEK
VDKYR	Non	Non	−6.02	11	9	679.8	VDK-Y-R
VQQEL	Non	Non	−6.61	9	10	615.7	VQQ-E-L
YDNHL	Non	Allergen	−4.08	10	11	660.7	Y-DNH-L
ADIKEL	Non	Allergen	−5.52	10	11	687.8	ADIK-E-L
AEMPPH	Non	Allergen	−3.76	7	10	680.8	Good
AINPYR	Non	Allergen	−3.75	10	9	732.8	AINPY-R
AVPDPG	Non	Allergen	−3.96	6	9	554.6	Good
CIAINP	Non	Non	−2.92	7	8	629.8	Good
DQIDQL	Non	Allergen	−5.88	11	13	730.8	Good
IIYSEF	Non	Allergen	−0.70	9	9	754.9	IIY-SE-F
NKVKPL	Non	Allergen	−5.18	9	8	697.9	NK-VKP-L
QCNPVL	Non	Allergen	−6.14	8	9	672.8	Good
QKVKSR	Non	Non	−5.41	12	9	687.8	QK-VK-SR
QMNPPK	Non	Non	−5.93	8	9	713.8	QM-NPPK

Table S6 Predicted toxicity, allergenicity, and intestinal epithelial permeability of newly formed peptides by gastrointestinal proteases

Peptide	Toxicity	Allergenicity	CLog*P*	H-bond donors	H-bond acceptors	MWT
ADIK	Non	Allergen	−6.01	7	7	445.5
AE	Non	Allergen	−3.76	4	5	218.2
AK	Non	Non	−3.49	4	3	217.3
ASK	Non	Non	−5.23	6	5	304.3
CEQ	Non	Non	−5.37	6	7	378.4
CINY	Non	Non	−2.61	7	7	511.6
DNH	Non	Non	−6.13	7	8	384.3
DNQI	Non	Allergen	−6.18	8	9	488.5
EH	Non	Allergen	−4.35	5	6	284.3
EM	Non	Allergen	−3.70	4	5	278.3
EN	Non	Allergen	−5.28	5	6	261.2
EP	Non	Allergen	−3.59	3	5	244.2
ESG	Non	Allergen	−5.47	6	7	291.3
ESL	Non	Non	−4.18	6	7	347.4
ET	Non	Allergen	−4.38	5	6	248.2
IIY	Non	Non	−1.41	5	5	407.5
IK	Non	Allergen	−3.27	4	3	259.3
NCW	Non	Non	−4.06	6	5	421.5
NDL	Non	Non	−4.31	6	7	360.4
NH	Non	Non	−5.36	5	5	269.3
NPM	Non	Non	−5.08	4	5	360.4
NPP	Non	Allergen	−4.42	3	5	326.3
NPPK	Non	Non	−4.91	5	6	454.5
NR	Non	Allergen	−5.59	6	4	288.3
PIY	Non	Allergen	−1.58	5	5	391.5
QDH	Non	Non	−5.77	7	8	398.4
QESK	Non	Allergen	−9.00	9	9	490.5
QM	Non	Non	−4.35	4	4	277.3
QQK	Non	Allergen	−6.07	7	6	402.4
QVK	Non	Allergen	−5.61	6	5	373.4
QY	Non	Non	−4.42	5	5	309.3
SE	Non	Allergen	−4.32	5	6	234.2
SI	Non	Non	−3.65	4	4	218.3
SK	Non	Allergen	−4.05	5	4	233.3
SR	Non	Allergen	−4.63	6	4	261.3
VDK	Non	Non	−5.93	6	6	360.4
VEK	Non	Non	−5.57	6	6	374.4
VKP	Non	Allergen	−3.49	4	4	342.4
VQQ	Non	Non	−6.54	6	6	373.4
VT	Non	Allergen	−3.66	4	4	218.3

Figure S1 Nine new ACE inhibitory peptides located in myosin heavy chain of *Todarodes pacificus*

1 MSSYDPSDPDMEFLCLTRQRQLEITTVPFDGKKNCWVPDPEQGFVSAEIQSTKGDEVTVK

61 TDKSMEMRTVKKDDVG | **QM** | **NPPK** | FEMNMDMANLTFLNEA | **SI** | LHNLRSRYESGFIYTYSGLF

121 CIAINPYRRLPIYTQGLVDKYRGKRRAEMPPHLF | **SI** | ADNAY | **QY** | MLQDHENQSMLITGESG

181 AGKTENTKKVI | **QY** | FALVAASLTSGGKDKKKEEEKKKDEKKGTLEDQIVQCNPVLEAYGNA

241 KTTRNNNSSRFGKFIRIHFGTQGKIAGADIETYLLEKSRVTYQQSAERNYHIFYQLLSPA

301 FPANIEKILAVPDPGLYGFINQGHLSVDGIDDEEEMQLTDTAFDVLGFTDDEKLSMYKCT

361 GCILHLGEMKWKQRGEQAEADGTAEAEKVAFLLGVNAGDLLKCLLKPKIKVGTEYVTQGR

421 NKDQVSN | **SI** | AAL | **AK** | SLYDRMFNWLVKRVNTTLDTK | **AK** | RQFFIGVLDIAGFEIFDYNSFEQ

481 LCINYTNERLQQFF | **NH** | HMFVLEQEEYKKEGIVWEFIDFGLDLQACIELIEKPMGIL | **SI** | LE

541 EECMFPKASDLSFKNKLYD | **NH** | LGK | **NPM** | FGKPKPPKAGCIEAHFALHHYAGSVSYNISSWL

601 EKNKDPINENVVELLQTSKEPIVKMLFTPPEDTSPAGGKKKKGKSAAFQTISSTHKESLN

661 KLMKNLYSTHPHFVRCIIPNELKTPGMIDAALVLHQLRCNGVLEGIRICRKGFPNR | **IIY** | S

721 EFKQRY | **SI** | LAP | **NAIP** | SGFADGKVVTDKVLSALQLDTNEYRLGNTKVFFKAGVLGMLEDMR

781 DERLSKIISMFQAHIRGYLMRKAYKKLQDQRIGLTLIQRNIRKWLMLRNWEWWRLFNKVK

841 PLLGMARQEEENKKAAEEF | **AK** | MKEEFLRCE | **QM** | RKELEEQNTVLMQQKNDLVISMSSSDDA

901 IIESEEKIEGLIKQKSDMETHIKELEEKLMDEEDAATDLAA | **AK** | KKSEADIKELKQDVEDL

961 EAGL | **AK** | AEQEKTTKDNQIKTLQDEMANQDEALSKVNKEKKALEEVQKKTLEDLQAEEDKV

1021 | **NH** | LSKLKTKLEQTLDELEDNLEREKKIRADVDK | **AK** | RKVEQDLKTTQETVEDLERVKRDLE

1081 DAGRKKDMEINGLNSKLEDEQNLVAQLQKKIKELQARIEELEEELEAERQARSKVEKQRT

1141 ELSREMEELGERLDEAGGATAA | **QM** | ELNKKREQELLRLRRDLEENTMQNESTIATLRKKNQ

1201 EATNELGDQIDQLQKVKSRLEKEKNELKEKAEEAMMELKHREKNMGCSEKMSK | **QM** | EAQLS

1261 ELNGKIDEQAR | **SI** | SELGSQKSRLQVEAADLTRQLEEAEHNVGQLTKLKSSMGVNLED | **AK** | R

1321 SLEDEARLR | **AK** | LQAEVRNLNSDIDGIRESLEEEQESKTDLQRALSRANAEVQQWRSKFES

1381 EGAARADELYDSKRKLQAELSEAEQTADTLHSKCAALEK | **AK** | SRLQGELEDLAIDAERSSA

1441 HANNLEKKQRNFDKVVSEWQHKCNDLQAELENAQKEARSYSAELFRVRAQCEEVGDTVES

1501 LRRENKNLADEIHDLTDQLGEGGRSTHELEKARKRLALEKEELQAALEEAEAALEQEE | **AK** |

1561 VMRATLEISQIRQEIDRRLQEKEEEFDNTRR | **NH** | QRAIESMQASLEAE | **AK** | GKAEALRIKKK

1621 LEGDINELEIALDATNRGKAELEKNVKKYQGQIRELQSAVEEEQRQRDE | **AK** | EHFMMAERR

1681 CAAINGELEELRTILEQAERARKAAENELADASDRVNELTAQVSTSNSQKRKLEGDVTAM

1741 QSDLDELNNELKDADDR | **AK** | HAMGDATRLADELRQEQDHGL | **SI** | EKMRKSLESQVKELQVRL

1801 DESEAAALKGGKKMIQKLESRVRELEAELDSEQRRHAETQKSMRKVDRRVKELSFQQEED

1861 RKNYERMQELVDKLQNKIKTYKRQVEEAEEIAAINL | **AK** | FRKVQQELEDAEERADQSEGAL

1921 QKLR | **AK** | NRSSVSVARTSPM

Figure S2 Regional flexibility (green) of ACE with and without peptides Ile-Ile-Tyr and Asn-Pro-Pro-Lys during molecular dynamics simulation.

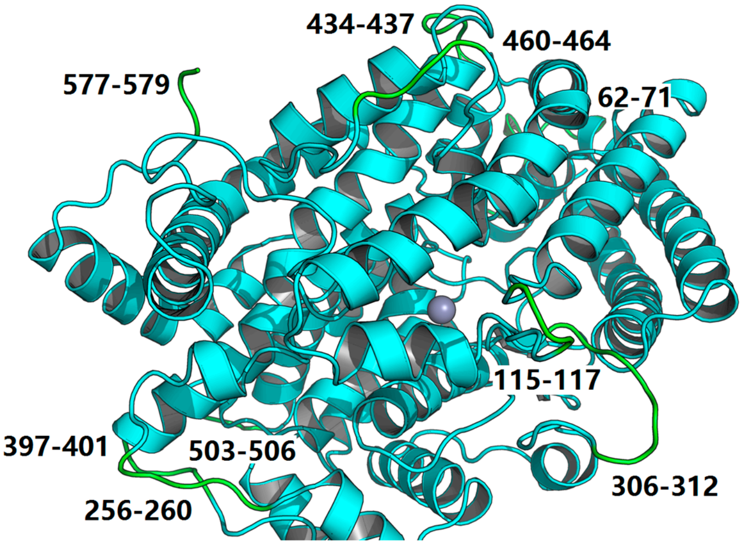

Supplement: Supplementary file 1 [file ijms-20-04159-s001.pdf]
